# Implications of Advancing Paternal Age: Does It Affect Offspring School Performance?

**DOI:** 10.1371/journal.pone.0024771

**Published:** 2011-09-21

**Authors:** Anna C. Svensson, Kathryn Abel, Christina Dalman, Cecilia Magnusson

**Affiliations:** 1 Division of Public Health Epidemiology, Department of Public Health Sciences, Karolinska Institutet, Stockholm, Sweden; 2 Centre for Women's Mental Health, Manchester Academic Health Sciences Centre, University of Manchester, Manchester, United Kingdom; Hôpital Robert Debré, France

## Abstract

Average paternal age is increasing in many high income countries, but the implications of this demographic shift for child health and welfare are poorly understood. There is equivocal evidence that children of older fathers are at increased risk of neurodevelopmental disorders and reduced IQ. We therefore report here on the relationship between paternal age and a composite indicator of scholastic achievement during adolescence, i.e. compulsory school leaving grades, among recent birth cohorts in Stockholm County where delayed paternity is notably common. We performed a record-linkage study comprising all individuals in Stockholm County who finished 9 years of compulsory school from 2000 through 2007 (n = 155,875). Data on school leaving grades and parental characteristics were retrieved from administrative and health service registers and analyzed using multiple linear regression. Advancing paternal age at birth was not associated with a decrease in school leaving grades in adolescent offspring. After adjustment for year of graduation, maternal age and parental education, country of birth and parental mental health service use, offspring of fathers aged 50 years or older had on average 0.3 (95% CI −3.8, 4.4) points higher grades than those of fathers aged 30–34 years. In conclusion, advancing paternal age is not associated with poorer school performance in adolescence. Adverse effects of delayed paternity on offspring cognitive function, if any, may be counterbalanced by other potential advantages for children born to older fathers.

## Introduction

The average age at childbearing has increased markedly in many high income countries [1] as a consequence of societal changes, such as increased labor force participation of women. This demographic shift is likely to have public health consequences for child health and welfare. While the potential offspring risks and gains of delaying maternity have been meticulously researched, debated and subject to policy action [2]–[5], the consequences of a similar demographic shift in paternal age has received far less attention.

Over the past years, increasing paternal age has been linked with a range of rare congenital syndromes in the offspring [6]–[8]. There is also growing evidence that advancing paternal age is a risk factor for several neurodevelopmental disorders including autism [9], bipolar disorder [10] and schizophrenia [11]. More recently, associations between paternal age and measures of child and adolescent intelligence quotient (IQ) [12], [13] have been reported and received extensive media attention [14].

**Figure 1:**
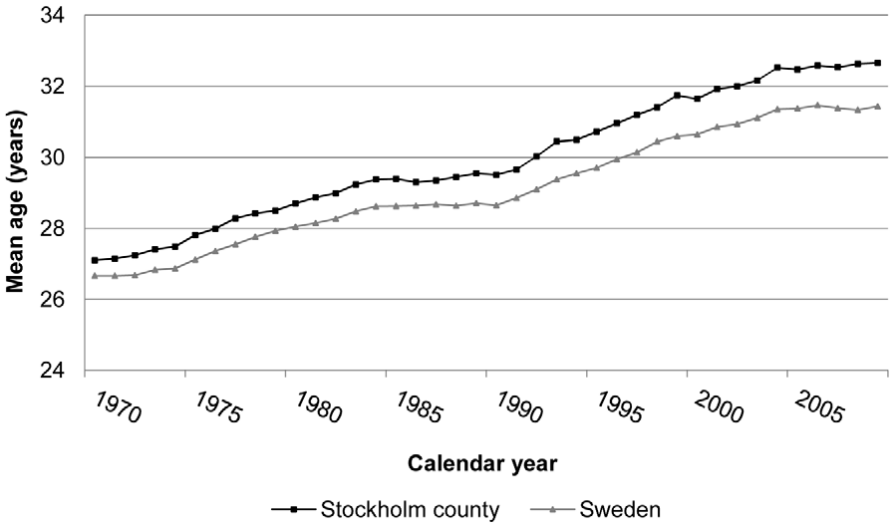
Trends in paternal mean age at first child birth in Sweden and Stockholm County, 1970–2009 [**20**].

Scholastic achievement is important as a predictor of future educational and occupational success [15] and is strongly linked to health in later life [16], [17]; hence it is a highly relevant outcome for appraisal of the net impact of current trends in timing of fatherhood on child welfare. Furthermore, school performance is highly correlated with IQ [18], [19] and hence of particular interest in light of the proposed negative influence of advancing paternal age on cognitive function [12], [13]. We, therefore, report here on the relationship between paternal age and compulsory school leaving grades from a register-based total population study set in Stockholm County, where the average paternal age is particularly high [20] (Figure 1).

## Materials and Methods

### Ethics statement

The study has been approved by the ethical review board (Dnr 2007/478-31).

### Study population and design

We performed a record-linkage study comprising all individuals in Stockholm County who completed their 9 years of compulsory schooling between 2000 and 2007 (n = 155,875). To prevent confounding by migrant status and to minimize missing data, we excluded individuals born outside of Sweden (n = 9,688). In the statistical analysis, children with missing data on any of the covariates (n = 9,367) were excluded. Thus, the final data set included 136,820 individuals. The research ethics committee at Karolinska Institutet, Stockholm, provided ethics approval.

### Data sources

We linked various administrative and health service registers, using the unique national registration number assigned to each resident of Sweden.

#### Swedish national school register

The Swedish national school register contains school grades for pupils graduating from the final year of compulsory education since 1988. The quality of the data in the National School Register is high and summary statistics are published on a regular basis [21].

In Sweden, compulsory schooling lasts 9 years and school attendance is compulsory for all children between the ages of 7 and 16. The majority (over 90 percent of the pupils), attend municipal compulsory schools, a smaller proportion attend independent schools and less than one percent go to international schools and national boarding schools.

Independent schools are included in the school register from 1993 onwards, but pupils attending schools applying alternative educational methods, for instance Waldorf schools, might receive special types of leaving certificates [22].

In the spring of 1998, Sweden introduced a target and knowledge-related grading system. There is a nationally determined syllabus for each subject with goals for the educational attainment within the subject. Final grades are assigned on the basis of these targets with the aid of adopted grading criteria. Before selection for upper secondary schools, the pupil's final grade is calculated as the sum of the points of the pupil's 16 best grades in the leaving certificate. Grades are awarded on a three-grade scale: Pass is counted as 10 merit points; Pass with Distinction as 15 and Pass with Special Distinction as 20 merit points. The maximum merit rating is 320 points.

#### Additional Registers

The *Swedish Multi-Generation Register* includes all individuals born in Sweden since 1932 and ever registered as living in Sweden after 1960, and their biological parents. [23]. Biological parents and their date of birth were identified through this source. Country of birth and parental educational level was retrieved from the *Education-* [24] and *Population Registers* [25], respectively. Parental psychiatric service use was assessed via the *Stockholm County Council VAL Database*, an administrative register covering all publically financed health services in Stockholm county since 1990, supplemented by inpatient data from the *Swedish Hospital Discharge Register* which contains details on virtually all hospitalisations in Sweden from 1973 [26].

### Statistical analyses

We conducted linear regression analyses to estimate crude and adjusted mean differences in school leaving grades and their 95% intervals in relation to paternal age. We used the PROC GENMOD procedure in SAS version 9.1, employing the identity link. Paternal and maternal ages were categorized into 5-year intervals. Fathers aged 30 to 34 years at the offspring's birth comprised the largest category and therefore served as the reference group. To control for confounding by socioeconomic status, we used highest achieved maternal and paternal education, as it is the most stable socioeconomic variable over time [27]. Furthermore, we adjusted for maternal age and parental country of birth, parental psychiatric service use and offspring's year of graduation in order to control for possible confounding factors. The following 3 models were used: (1) crude analyses of the influence of paternal age, (2) analyses of paternal age adjusted for maternal age, (3) analyses of paternal age adjusted for maternal age, highest achieved maternal and paternal education completed by 2006, maternal and paternal country of birth, any maternal and paternal psychiatric service use and offspring's year of graduation. Variables were categorized as demonstrated in Table 1. We did not adjust for obstetric complications as they are unlikely to meet definition of confounders, but may mediate the relationships, and therefore, including them may result in biased estimates [28].

**Table 1:** Distribution of compulsory school leaving grades in relation to parental characteristics.

Parental characteristics	N	Mean (SD)	Mean	95% CI
**Paternal age (years)**			difference	
<20	985	167.2 (69.7)	−55.2	−59.1, −51.3
20–24	15,201	190.9 (63.8)	−31.5	−32.7, −30.4
25–29	40,865	210.4 (62.6)	−12.0	−12.8, −11.2
30–34	45,743	222.4 (61.4)	ref	
35–39	27,383	224.2 (61.4)	1.8	0.9, 2.8
40–44	11,298	222.9 (62.3)	0.5	−0.8, 1.8
45–49	3,416	220.3 (64.0)	−2.1	−4.2, 0.1
50+	1,296	216.4 (66.2)	−6.0	−9.4, −2.5
**Maternal age (years)**				
<20	3,576	174.0 (66.5)	−52.0	−54.1, −49.9
20–24	28,405	196.1 (63.7)	−29.8	−30.8, −28.9
25–29	50,519	216.0 (62.0)	−10.0	−10.8, −9.2
30–34	42,146	226.0 (60.7)	ref	
35–39	17,953	227.8 (60.1)	1.8	0.7, 2.9
40–44	3,477	227.5 (61.8)	0.5	−1.7, 2.6
45+	111	213.2 (73.9)	−12.8	−24.3, −1.3
**Paternal educational level** *				
nine-year compulsory school, not finished	5,050	181.7 (67.6)	ref	
nine-year compulsory school	19,874	186.8 (64.4)	5.1	3.3, 6.9
upper secondary school, 2 years	41,930	199.1 (60.9)	17.4	15.7, 19.1
upper secondary school, 3 years	20,199	221.3 (57.8)	39.6	37.8, 41.4
university education shorter than three years	20,410	231.8 (54.2)	50.1	48.3, 51.8
university education three years or more	28,256	249.9 (49.9)	68.2	66.5, 69.9
post-graduate	3,123	263.6 (46.6)	81.3	79.2, 84.4
Missing	7,345	199.1 (70.2)		
**Maternal educational level** *				
nine-year compulsory school, not finished	3,936	178.1 (68.0)	ref	
nine-year compulsory school	14,125	180.2 (66.8)	2.1	−0.02, 4.1
upper secondary school, 2 years	44,883	197.1 (61.6)	18.9	17.0, 20.8
upper secondary school, 3 years	20,990	217.5 (58.6)	39.3	37.3, 41.3
university education shorter than three years	25,280	232.0 (54.6)	53.9	51.9, 55.9
university education three years or more	33,199	245.8 (52.2)	67.6	65.7, 69.6
post-graduate	1,368	266.1 (43.9)	88.0	84.4, 91.6
Missing	2,406	201.2 (69.8)		
**Year of graduation**				
2000	14,534	213.5 (61.4)	ref	
2001	15,659	212.8 (63.9)	−0.7	−2.1, 0.7
2002	16,700	213.7 (63.4)	0.2	−1.2, 1.6
2003	17,155	214.4 (63.8)	0.9	−0.5, 2.3
2004	18,919	216.2 (63.5)	2.7	1.3, 4.0
2005	19,866	217.3 (62.5)	3.8	2.4, 5.1
2006	21,710	217.7 (62.8)	4.1	2.8, 5.5
2007	21,677	217.7 (63.8)	4.1	2.8, 5.5
**Any psychiatric admission mother**				
No	101,074	221.7 (60.2)	ref	
Yes	45,113	201.1 (67.5)	−19.6	−20.3,−18.9
**Any psychiatric admission father**				
No	116,385	219.5 (61.3)	ref	
Yes	29,802	200.9 (68.0)	−18.5	−19.3, −17.7
**Maternal country of birth**				
Sweden	117,216	218.0 (62.1)	ref	
Nordic countries**	9,649	207.0 (67.3)	−11.0	−12.3, −9.7
Outside Nordic countries	19,322	206.6 (66.2)	−12.4	−13.4, −11.5
**Paternal Country of birth**				
Sweden	115,998	219.0 (61.7)	ref	
Nordic countries**	6,531	197.9 (68.8)	−21.1	−22.7, −19.6
Outside Nordic countries	23,653	204.3 (66.5)	−14.6	−15.5, −13.8

* Parental education categorized according to the standard system used by Statistics Sweden.

** Denmark, Finland, Iceland and Norway, not including Sweden.

Earlier research indicates that age of the father at the birth of his first child, rather than his age at the birth *per se*, constitutes a risk factor for schizophrenia [29]. Hypothesizing that factors associated with selection into late fatherhood might attenuate any positive effects of advancing paternal age, we also performed a restricted analysis of fathers younger than 40 years of age at the birth of their first child. If factors leading to delayed age at first fatherhood also have deleterious effects on offspring school performance, removal of this possible confounder would be expected to generate an improvement in school performance among offspring of older fathers. The cut-off for paternal age was based on studies showing that major changes in offspring risk of neurodevelopmental disorders tend to occur after this age [9]–[11].

In an exploratory post hoc analysis, an interaction term between paternal age and offspring sex was included in the models, to test whether any effect of paternal age on school grades was modified by the sex of the child. Statistically significant differences were assumed when p<0.05 (two-sided).

## Results

Table 1 shows the distribution of grades in school leaving certificates in relation to parental characteristics. Individuals born to older or better educated parents, parents with no records of psychiatric admissions or to parents of Swedish origin, on average had higher grades in crude comparisons. The grades increased somewhat with year of graduation and were higher for girls (mean = 227, SD = 62) than for boys (mean = 207, SD = 61).

**Figure 2:**
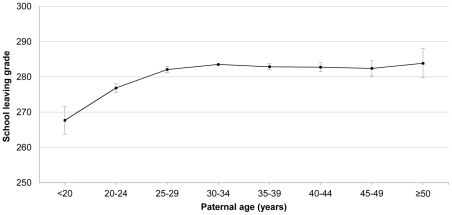
Adjusted^*^ mean compulsory school leaving grades with 95% confidence intervals in relation to paternal age. ^*^ Adjusted for maternal age, maternal and paternal educational level, mental health service use, country of birth and year of graduation of the child.

The results from the main analyses are presented in Table 2 (upper panel) and Figure 2. Overall, advanced paternal age was not associated with reduced offspring school leaving grades in either the crude or the adjusted analyses. There was, however, a clear trend of diminishing grades with decreasing paternal age below 30 years. Offspring born to teenage fathers had on average 53.8 (95% CI 57.9, 49.8) points lower grades, than the reference group (aged 30 to 34 years) in crude analyses. This effect of young paternity was sharply attenuated, but remained statistically significant, after adjustment for covariates with an especially profound impact of adjustment for parental level of education.

**Table 2:** Average compulsory school leaving grades in relation to paternal and maternal age.

Full sample (n = 136,820)						
	Model 1		Model 2*		Model 3**	
Variable	Estimate	95% CI	Estimate	95% CI	Estimate	95% CI
Paternal age (y)						
<20	−53.8	−57.9, −49.8	−26.1	−30.4, −21.8	−15.8	−19.7, −11.9
20–24	−31.7	−32.9, −30.6	−14.8	−16.1, −13.4	−6.6	−7.9, −5.4
25–29	−12.2	−13.0, −11.3	−5.2	−6.1, −4.3	−1.4	−2.2, −0.6
30–34	reference		reference		reference	
35–39	2.0	1.0, 2.9	−1.3	−2.3, −0.3	−0.7	−1.5, 0.2
40–44	1.8	0.4, 3.1	−2.7	−4.1, −1.3	−0.8	−2.1, 0.5
45–49	−0.5	−2.8, 1.8	−5.1	−7.4, −2.7	−1.1	−3.3, 1.1
≥50	−1.3	−5.8, 3.2	−5.9	−10.4, −1.4	0.3	−3.8, 4.4

Paternal age at first birth less than 40 years (n = 133,645)						
	Model 1		Model 2*		Model 3**	
Variable	Estimate	95% CI	Estimate	95% CI	Estimate	95% CI
Paternal age (y)						
<20	−53.8	−57.9, −49.8	−26.1	−30.4, −21.8	−16.8	−20.8, −12.9
20–24	−31.7	−32.9, −30.6	−14.8	−16.1, −13.4	−7.2	−8.4, −5.9
25–29	−12.2	−13.0, −11.3	−5.2	−6.1, −4.3	−1.6	−2.4, −0.7
30–34	reference		reference		reference	
35–39	2.0	1.0, 2.9	−1.3	−2.3, −0.3	−0.8	−1.7, 0.1
40–44	−1.8	−3.3, −0.4	−6.5	−8.1, 5.0	−3.3	−4.7, −1.8
45–49	−4.7	−7.4, −2.1	−9.5	−12.2, −6.8	−3.5	−6.0, −1.0
≥50	−1.3	−6.4, 3.9	−6.3	−11.5, −1.3	1.5	−3.2, 6.2

* Adjusted for maternal age.

** Additionally adjusted for maternal and paternal educational level, psychiatric service use, country of birth and year of graduation of the child.

In additional analyses which aimed to account for selection into late fatherhood, we restricted the sample to offspring of fathers that were younger than 40 years at the birth of their first child. Results from these analyses were similar to the main analyses (Table 2, lower panel). The association between paternal age and school leaving grades did not vary appreciably with offspring sex (results not shown).

## Discussion

In this total population study from Stockholm County comprising more than 135,000 youths, we found no relationship between advancing paternal age and poorer offspring school performance. This null finding was consistent in crude and adjusted models. On the contrary, offspring of fathers who were under 35 years of age had on average somewhat lower grades in school leaving certificates. Adjustment for highest achieved parental education greatly reduced the negative effect of young paternity on school performance, but a minor reduction persisted.

Parental age at first birth is reaching an all-time high in many parts of the world [1]. This phenomenon is especially pronounced in Sweden, in general, and in Stockholm County, in particular, where the average paternal age at first birth has increased from 27 years in 1970 to almost 33 years today (Figure 1) [20]. Advancing paternal age may provide for offspring risks as well as benefits; and these could be mediated through biological and/or psychosocial mechanisms. Advancing paternal age has been linked to a range of neurodevelopmental [9]–[11] and congenital disorders [6]–[8] as well as decreased IQ [12], [13], [30], [31]. However, the net effect of delayed fatherhood on child welfare and health has yet to be assessed. Rearing by older fathers might offer advantages that enhance offspring social and cognitive functioning, e.g. an American study found that older fathers spent more time with their children and provided better financial security [32].

In contrast to some [12], [13], [30], [31], but not all [33] earlier studies of paternal age and cognitive outcomes, we found no evidence of a decline in school performance among offspring born to older fathers. In healthy men, germ cells divide continuously and the risk of errors in DNA transcription increases exponentially with age [34]. It has been suggested that the association between paternal age and adverse outcomes are due to de novo mutations or epigenetic effects caused by ageing sperm cells [12],[13]. Previous studies have attempted to establish how general measures of IQ [12], [13], [30], [31] are influenced by paternal age, but the results are inconsistent. It has been suggested that the pattern of the relationship is best described by an inverted U-shaped curve, where the cognitive function in offspring increases with paternal age up to about thirty years and thereafter decreases [12], [30], [31]. Others have found a close to linear association between increasing paternal age and declining cognitive performance [13]. Neither of these patterns of association are congruent with a mutation rate in human male germ cells which increases exponentially with age [34], [35].

An alternative explanation is that confounding factors associated with delayed fatherhood, including personality traits, maternal age or level of education, rather than advancing paternal age *per se*, produce the association [29], [33]. Indeed, some older studies were not able to account for the potentially confounding effects of socioeconomic or other family level factors [30], [31], in particular parental education. Reanalyzing data from an earlier study [13], Edwards *et al.* [33] reported that much of the effect of paternal age on neurocognitive outcomes disappeared when they adjusted for family characteristics, including some intermediate variables (i.e. factors affected by the exposure), such as sibship size and birth weight. However, these results are susceptible to *collider-stratification bias*, as the estimated indirect effect removed by adjusting for a mediator can itself be confounded if there are common causes between the mediator and the outcome [28]. In a recent study, Peterson *et al.* [29] reported an association between selection into late fatherhood and schizophrenia. Thus, we hypothesized that extant social cognitive and/or genetic reasons leading to delayed first-time fatherhood would have a negative influence on children's school performance, rather than de novo mutations at the time of conception and thus attenuate any potential positive effects provided by older fathers. However, restriction to younger first-time fathers did not inflate school performance scores in offspring of older fathers.

It is also possible that contextual differences and discrepancies in outcome measures may explain inconsistencies between studies. There is an extensive literature investigating the overlap between intelligence and educational achievement [18], [19], but academic outcomes are influenced by numerous factors, such as school and family environment [36], [37] and personality traits [38], [39]. Our null findings, therefore, may indicate that the rearing environment provided by older fathers compensates for any subtle neurocognitive impairments in offspring, meaning that any such effects are not translated into reduced educational attainment. But they also support the notion that advancing paternal age is not associated with a reduced level of cognitive performance consistent with some other studies of paternal age and neurocognitive or developmental outcome (e.g. [33]).

We found that offspring of younger fathers had somewhat lower school leaving grades than offspring of those aged more than thirty years; and to a large extent, this relationship was explained by level of parental education. Earlier research also suggests that offspring of younger fathers perform less well on cognitive tests [12], [13], [30], [31]. Our results indicate that much of the previously reported effect could be explained by confounding factors in general, and by parental level of education in particular [30], [31].

There are limitations to consider in this study. First, we only included data from pupils in the regular school system. Children with severe intellectual disabilities are not included in the National School Register and hence are not in our study population. Moreover, we acknowledge the challenge to account fully for parental socioeconomic position while avoiding overadjustment for intermediate variables. Socioeconomic status can be a consequence, rather than a cause, of parental age at parenthood [40], [41], i.e. it can act both as a confounder and a mediator in the relationship between parental age and offspring scholastic achievement. Further, many socioeconomic indicators are inherently age dependent, e.g. level of current income tends to increase throughout one's occupational career [27]. We chose parental education as an indicator of SES as it is a fairly stable socioeconomic variable over time. Moreover, parental education was measured at least fifteen years after offspring birth, allowing young parents at least fifteen years of catch-up time to fulfill their intrinsic potential and minimizing the impact of age at parenthood on education. In addition to socioeconomic status, we adjusted for parental psychiatric service use to reduce further confounding by genetic liability for psychiatric disorder and/or cognitive impairment, but obviously many other unmeasured genetic and environmental factors might also influence the association between paternal age and school performance.

We conclude that advancing paternal age is not associated with poorer school performance measured in adolescents. Adverse effects of delayed paternity on offspring cognitive function in the normal IQ range, if any, maybe counterbalanced by social and family environmental advantages for children born to older fathers. Appraising the public health effects of delayed paternity is an important area for further research; at least our null findings are reassuring for the increasing number of men who are postponing fatherhood.
